# Germline mutations in *RYR1* are associated with foetal akinesia deformation sequence/lethal multiple pterygium syndrome

**DOI:** 10.1186/s40478-014-0148-0

**Published:** 2014-12-05

**Authors:** Arthur B McKie, Atif Alsaedi, Julie Vogt, Kyra E Stuurman, Marjan M Weiss, Hassan Shakeel, Louise Tee, Neil V Morgan, Peter G J Nikkels, Gijs van Haaften, Soo-Mi Park, Jasper J van der Smagt, Marianna Bugiani, Eamonn R Maher

**Affiliations:** Department of Medical Genetics, University of Cambridge and NIHR Cambridge Biomedical Research Centre, Cambridge Biomedical Campus, Cambridge, CB2 0QQ UK; Centre for Rare Diseases and Personalised Medicine, University of Birmingham, Edgbaston, Birmingham, B15 2TT UK; West Midlands Regional Genetics Service, Birmingham Women’s Hospital, Birmingham, B15 2TG UK; Department of Clinical Genetics, VU University Medical Center, Amsterdam, The Netherlands; Department of Clinical Genetics, Addenbrooke’s Treatment Centre, Cambridge University Hospitals NHS Foundation Trust, Hills Road, Cambridge, CB2 0QQ UK; Department of Medical Genetics, University Medical Center Utrecht, University of Utrecht, Utrecht, The Netherlands; Department of Pathology, VU University Medical Center, Amsterdam, the Netherlands; Department of Pathology, University Medical Center Utrecht, Utrecht, The Netherlands; Department of Medical Genetics, School of Clinical Medicine, University of Cambridge, Addenbrooke’s Treatment Centre, Cambridge Biomedical Campus, Box 238, Cambridge, CB2 0QQ United Kingdom

**Keywords:** Multiple pterygium syndrome, Foetal akinesia, *RYR1* mutations, Myopathy

## Abstract

**Introduction:**

Foetal akinesia deformation sequence syndrome (FADS) is a genetically heterogeneous disorder characterised by the combination of foetal akinesia and developmental defects which may include pterygia (joint webbing). Traditionally multiple pterygium syndrome (MPS) has been divided into two forms: prenatally lethal (LMPS) and non-lethal Escobar type (EVMPS) types. Interestingly, FADS, LMPS and EVMPS may be allelic e.g. each of these phenotypes may result from mutations in the foetal acetylcholine receptor gamma subunit gene (*CHRNG*). Many cases of FADS and MPS do not have a mutation in a known FADS/MPS gene and we undertook molecular genetic studies to identify novel causes of these phenotypes.

**Results:**

After mapping a novel locus for FADS/LMPS to chromosome 19, we identified a homozygous null mutation in the *RYR1* gene in a consanguineous kindred with recurrent LMPS pregnancies. Resequencing of *RYR1* in a cohort of 66 unrelated probands with FADS/LMPS/EVMPS (36 with FADS/LMPS and 30 with EVMPS) revealed two additional homozygous mutations (in frame deletions). The overall frequency of *RYR1* mutations in probands with FADS/LMPS was 8.3%.

**Conclusions:**

Our findings report, for the first time, a homozygous *RYR1* null mutation and expand the range of *RYR1*-related phenotypes to include early lethal FADS/LMPS. We suggest that *RYR1* mutation analysis should be performed in cases of severe FADS/LMPS even in the absence of specific histopathological indicators of *RYR1*-related disease.

## Introduction

Foetal akinesia deformation sequence syndrome (FADS) is characterised by a variable combination of foetal akinesia, prenatal growth restriction, developmental defects (including cleft palate, cryptorchidism, cystic hygroma, heart abnormalities, intestinal malrotation and lung hypoplasia), arthrogryposis and, in some cases, limb pterygia, so that there is phenotypic overlap between FADS and severe cases of multiple pterygium syndrome (MPS) [1]. Clinically MPS can be divided into the severe lethal form (LMPS) and the milder non-lethal Escobar type (EVMPS). MPS is most commonly inherited as an autosomal recessive trait though autosomal dominant and X-linked cases are described [2-4]. Both MPS and FADS are genetically heterogeneous and although, in some cases, a diagnosis of a specific primary myopathy, metabolic or neurodevelopmental disorder can be made by clinical and pathological investigations, the underlying aetiology is unknown in the majority of cases [5]. Previously, we and others have reported that germline mutations in genes encoding specific components of the acetylcholine receptor (AChR) complex at the neuromuscular junction may present with autosomal recessively inherited forms of FADS, LMPS and EVMPS [6,7]. Thus mutations in *CHRNG* (which encodes the foetal gamma subunit of the acetylcholine receptor) have been associated with FADS, LMPS and EVMPS and mutations in genes that encode other subunits that make up the foetal acetylcholine receptor (*CHRNA1*, *CHRND*) or regulators of AChR function (e.g. *RAPSN* and *DOK7*) have been described in FADS/LMPS [8-10]. Interestingly, mutations in *CHRNA1*, *CHRND*, *RAPSN* and *DOK7* can also cause congenital myasthenia syndrome (CMS), a milder disorder that is characterised by muscle fatigability and, rarely, arthrogryposis [11-13].

Identification of the underlying genetic cause of FADS/MPS facilitates clinical management by providing (a) precise genetic diagnosis, (b) enabling accurate predictions of recurrence risk and prognosis and (c) allowing the possibility of prenatal diagnosis. However, FADS and MPS are genetically heterogeneous and in many cases mutations in acetylcholine receptor-related genes cannot be identified. In order to characterise potential genetic causes of FADS/MPS in such cases, we undertook molecular genetic investigations in cohorts of FADS, LMPS and EVMPS families that were enriched for autosomal recessively inherited forms of these disorders (i.e. enriched for parental consanguinity) and identified loss of function *RYR1* mutations as a cause of early lethal FADS/LMPS.

## Material and methods

### Patients

66 families with features of FADS/LMPS/EVMPS and no known underlying genetic cause were investigated. In 36 families their clinical phenotype was FADS/LMPS and in 30 the phenotype was EVMPS. Consanguinity was recorded in 48% of the FADS/LMPS families and 20% of the EVMPS families. All families gave informed consent, the study was approved by the South Birmingham Research Ethics Committee and performed in accordance with the ethical standards laid down in the 1964 Declaration of Helsinki [14].

### Molecular genetic analysis

#### Linkage analysis

**Figure 1:**
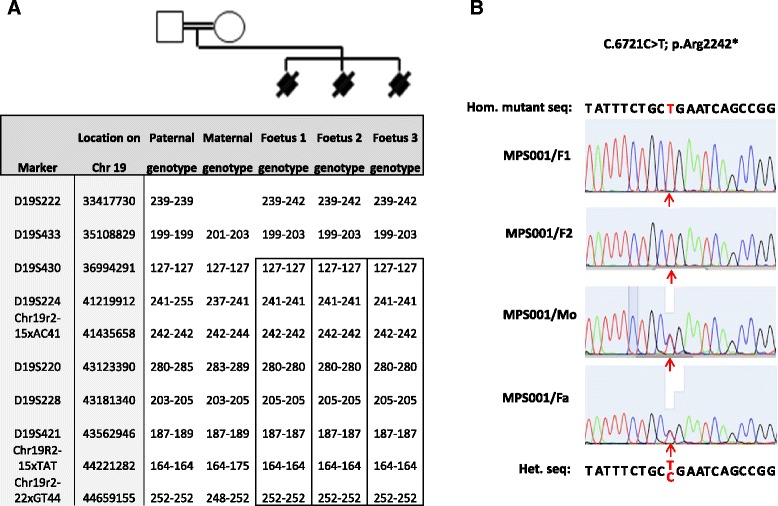
**A: Mapping of a consanguineous family (MPS001) with lethal multiple pterygium syndrome** **to** ***RYR1*** **.** The three affected foetuses shared a common homozygous region between 28,725,890 - 44,669,155 on chromosome 19. **B**: Chromatograms showing nonsense mutation (C.6721C > T; p.Arg2242*) at exon 42 in two affected foetuses (F1 and F2) and (in heterozygous state) in the parents (Mo and Fa).

A genome-wide linkage scan was carried out using the Affymetrix 250 K Human SNP Array 5.0 on DNA from stored foetal material of two affected siblings from a consanguineous family affected with FADS/LMPS. This scan excluded linkage to known FADS/LMPS genes and an ~10 Mb prime candidate region on chromosome 19 was identified and further evaluated by typing the parents and DNA from three affected foetuses with microsatellite markers (details on request and see Figure 1A).

#### RYR1 sequencing

*RYR1* gene sequencing was performed after amplification of all 106 coding exons. Initially, sequencing was performed on whole genome amplified DNA (Qiagen REPLI-g kits) and candidate variants were then confirmed on stock DNA samples. Flanking primers were designed from genomic sequence 20–80 nucleotides upstream or downstream from encoding exons. PCR products were sequenced in forward and reverse orientations using standard BigDye^R^ Terminator v3.1 cycle sequencing. Details of primer sequences are available on request. Sequence traces from each of the DNAs analysed was compared to the reference sequence from the ENSEMBL database (GRCh37:CM000681.1 - NM_000540; transcript ENST00000355481). The segregation of sequence variants was checked in other family members (when available) by BigDye^R^ Terminator v3.1 sequencing. Frequency information for RYR1 variants was sought from the NHLBI Exome variant server [http://evs.gs.washington.edu/EVS/] if available and the prediction of possible effects of any amino acid substitution was achieved with the PolyPhen-2 tool [http://genetics.bwh.harvard.edu/pph2/].

### Histopathological analysis

Histopathological analysis was performed on tissue obtained at autopsy from two fetuses of family MPS001 (12 + 6 and 14 + 0 weeks GA, respectively) and two age-matched controls (13 + 0 and 13 + 4 weeks GA, respectively) retrieved from the autopsy archive of the VU University Medical Center, Amsterdam, The Netherlands. sixum thick formalin-fixed paraffin-embedded tissue sections were processed according to standard protocols [15]. Histochemical staining included Hematoxylin & Eosin, Gomori trichrome and alizarin red S for calcium. After heat-induced antigen retrieval in 0.01 M citrate buffer (pH6), immunohistochemical staining was performed with antibodies against desmin (Abcam, 1:500), myosin heavy chain slow (Abcam, 1:100), active caspase 3 (Dako, 1:500), CD3 (Dako, 1:250), CD20 (Dako, 1:50) and CD45 (Dako, 1:100). Immunoreactivity was detected with 3,3′-diaminobenzidine as chromogen. Tissue sections were photographed using a Leica DM6000B microscope (Leica Microsystems). Omitting primary antibodies yielded no significant staining.

Ultrastructural analysis was performed on muscle tissue retrieved from formalin fixed material. The tissue was deparaffinised in xylene (60 minutes at 70°C), rehydrated, fixed with 2% glutaraldehyde in 0.1 M sodium cacodylate buffer (pH 7.4), post-fixed in 2% osmium tetroxide and embedded in epoxy resin. Ultrathin sections were stained with uranyl acetate and lead citrate and viewed in a FEI Technai 12 electron microscope.

The pictures were acquired as TIFF files and images were optimized for brightness and contrast using Photoshop, version 7.0 (Adobe systems, San Jose, CA).

## Results

### Clinical and molecular genetic analysis in MPS001 family

Genome wide linkage analysis was performed on a consanguineous family from The Netherlands (see Family MPS001 in Table 1) that had had six pregnancies affected by FADS/MPS. The proband (F2) presented was the second pregnancy that was terminated at 12 + 6 weeks of gestation because of an increased nuchal translucency of 9 mm, fetal akinesia and joint contractures. There was no intrauterine growth restriction. At post mortem examination there was a cystic hygroma, lung hypoplasia, webbing of both elbows and knees and arthrogryposis. The calvaria were absent due to birth trauma however there were no skeletal abnormalities (histopathology results are described below). In the family history, the first pregnancy was terminated because of increased nuchal translucency of 12 mm, fetal akinesia and a unilateral club foot. Additionally, the third, fourth, sixth and seventh pregnancy were terminated, because of increased nuchal translucency and fetal akinesia. The fifth pregnancy ended in an early miscarriage.

**Table 1:** **Clinical features of 66 probands in which** ***RYR1*** **mutation analysis was performed**

**Family number**	**Phenotype**	**Ethnicity**	**Consang.**
MPS001	LMPS	White	Y
MPS002	LMPS	South Asian	Y
MPS003	LMPS	Middle Eastern	Y
MPS004	LMPS	South Asian	Y
MPS005	LMPS	South Asian	Y
MPS006	LMPS	South Asian	Y
MPS007	LMPS	Not available	N
MPS008	LMPS	White	N
MPS009	LMPS	White	N
MPS010	FADS	Middle Eastern	Y
MPS011	LMPS	Not recorded	Y
MPS012	LMPS	North African	Y
MPS013	LMPS	White	N
MPS014	LMPS	South Asian	Y
MPS015	LMPS	White	N
MPS016	LMPS	Not available	Y
MPS017	LMPS	North African	Y
MPS018	FADS	White	N
MPS019	LMPS	White	N
MPS020	LMPS	White	N
MPS021	LMPS	South Asian	Y
MPS022	FADS	Middle Eastern	Y
MPS023	LMPS	Middle Eastern	Y
MPS024	LMPS	White	N
MPS025	FADS	Mixed race	N
MPS026	FADS	White	N
MPS027	LMPS	Not available	Y
MPS028	LMPS	Not available	N
MPS029	FADS/LMPS	Not available	N
MPS030	FADS/LMPS	Not available	N
MPS031	LMPS	White	N
MPS032	LMPS	White	N
MPS033	LMPS	White	N
MPS034	EVMPS	Not available	Y
MPS035	EVMPS	South Asian	Y
MPS036	EVMPS	White	N
MPS037	EVMPS	Not available	N
MPS038	EVMPS	White	N
MPS039	EVMPS	White	N
MPS040	EVMPS	White	N
MPS041	EVMPS	White	N
MPS042	EVMPS	White	N
MPS043	EVMPS	South Asian	N
MPS044	EVMPS	South American	N
MPS045	EVMPS	South Asian	Y
MPS046	EVMPS	South Asian	Y
MPS047	EVMPS	White	N
MPS048	EVMPS	African	Y
MPS049	EVMPS	Not available	N
MPS050	EVMPS	White	N
MPS051	EVMPS	White	N
MPS052	EVMPS	White	N
MPS053	EVMPS	White	Y
MPS054	EVMPS	White	N
MPS055	EVMPS	White	N
MPS056	EVMPS	White	N
MPS057	EVMPS	South American	N
MPS058	EVMPS	White	N
MPS059	EVMPS	African	N
MPS060	EVMPS	White	N
MPS061	EVMPS	White	N
MPS062	EVMPS	White	N
MPS063	EVMPS	White	N
MPS064	LMPS	Middle Eastern	?
MPS065	LMPS	White	N
MPS066	LMPS	North African	Y

Clinical phenotype (FADS = Foetal Akinesia Deformation Sequence, LMPS = Lethal Multiple Pterygium Syndrome; EVMPS = Escobar Variant Multiple Pterygium Syndrome), ethnic origin and presence of parental consanguinity is recorded).

After genotyping of DNA from two affected foetuses (first and second foetuses) by high resolution Affymetrix SNP arrays (Genome-Wide Human SNP Array 5.0) a large homozygous region on chromosome 19 was selected for further analysis. Genotyping with microsatellite markers (see Table 2), was then performed on parental DNA samples and DNA from three affected pregnancies. The additional genotyping defined the candidate autozygous region as Chr19; 35108829–44484993) (see Figure 1A). A microsatellite marker (D8S373) that mapped within the only other candidate autozygous region >2 Mb was demonstrated to be heterozygous in two affected foetuses and so excluded linkage to the chromosome 8 candidate interval (data not shown). At the time of analysis the candidate region on chromosome 19 contained 345 known or predicted genes including the ryanodine receptor 1 (skeletal) (*RYR1*) gene (Chr19;38924340–39078204).

**Table 2:** **Microsatellite markers employed in Mapping of chromosome 19 candidate region in consanguineous family (MPS001)**

**Marker**	**Genomic position**	**Source**
D19S222	Chr.19-28,725,890-28,726,217 bp	http://rgd.mcw.edu/rgdweb/report/marker/main.html?id=1341900
D19S433	Doesn’t map to Chr.19 assembly	http://rgd.mcw.edu/rgdweb/report/marker/main.html?id=1298168
D19S430	Chr.19-32,302,451-32,302,741 bp	http://rgd.mcw.edu/rgdweb/report/marker/main.html?id=1657125
D19S224	Chr.19-35,493,932-35,494,196 bp	http://rgd.mcw.edu/rgdweb/report/marker/main.html?id=1336138
Chr19r2-15xAC41	Chr.19-41,435,658-43,123,394	ftp://ftp.broad.mit.edu/pub/human_STS_releases/
D19S220	cHR.19-34,8798,595-34,879,871 BP	http://rgd.mcw.edu/rgdweb/search/markers.html?term=D19S220&speciesType=1
D19S228	Chr.19-34,937,645-34,937,798 bp	http://rgd.mcw.edu/rgdweb/report/marker/main.html?id=1338234
D19S421	Chr.19-38,871,106-38,871,460 bp	http://rgd.mcw.edu/rgdweb/report/marker/main.html?id=1337182
Chr19R2-15xTAT	Chr.19-44,221,282-44,669,155	ftp://ftp.broad.mit.edu/pub/human_STS_releases/
Chr19r2-22xGT44	Chr.19-44,221,282-44,669,155	ftp://ftp.broad.mit.edu/pub/human_STS_releases/

### RYR1 mutation analysis

Sequencing of *RYR1* in an affected foetus (F2) from Family MPS001 revealed a homozygous *RYR1* nonsense mutation (NM_000540.2(RYR1): c.6721C > T; p.(Arg2241*)). Further analysis demonstrated that a second affected foetus (F1) was also homozygous for c.6721C > T, whereas both parents were heterozygous for the nonsense mutation (see Figure 1B). Subsequently DNA from the F3, F4, F6 and F7 were also shown to be homozygous for the mutation (data not shown). The *RYR1* c.6721C > T mutation was previously detected (rs200563280), in the heterozygous state in 1 of 6503 individuals genotypes listed in the exome variant server (http://evs.gs.washington.edu/EVS) (no homozygous genotypes were detected at an average read depth of 49). In order to assess the potential role of recessive *RYR1* mutations in FADS, and/or MPS we proceeded to screen a further 66 probands for germline RYR1 variants. Two further potential candidate homozygous mutations were detected, a novel in-frame deletion of 27 nucleotides (NM_000540.2(RYR1): c.2097_2123del p.(Glu699_Gly707del)) and an in-frame deletion of 3 nucleotides c.7043delGAG p.(Glu2347del) (rs121918596)) (see Table 3). Each of the candidate mutations were detected in the homozygous state in individuals with FADS/MPS.

**Table 3:** **Rare** ***RYR1*** **variants detected in families with FADS/LMPS phenotypes**

**Family ID**	**DNA change**	**Protein change**	**Genotype**	**Segregation in family?**
**MPS001**	c.6721C > T	p.Arg2241^STOP^	**Homozygous**	**Yes**
**MPS002**	c.2097_2123del	p.(Glu699_Gly707del)	**Homozygous**	**Yes**
**MPS003**	c.7043delGAG	p.(Glu2347del))	**Homozygous**	**Yes**

### Clinical and molecular genetic analysis in MPS002 family

**Figure 2:**
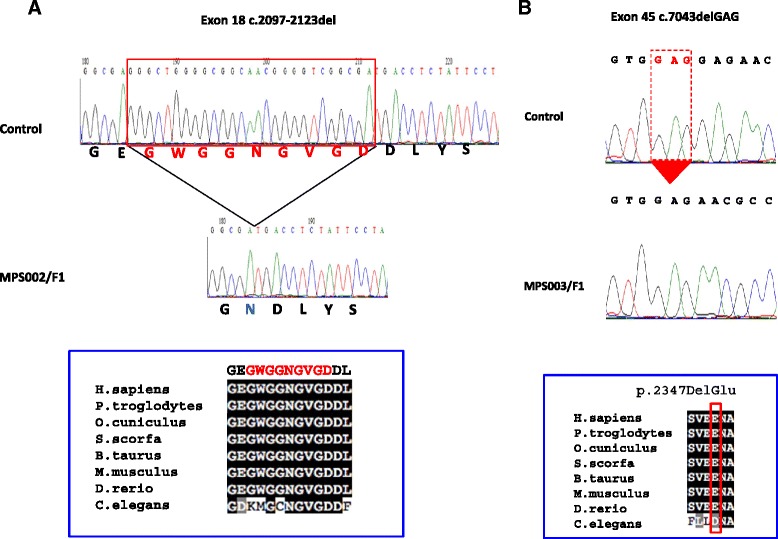
**Identification of RYR1 in frame deletions. A**. In the proband (F1) from Family MPS002 there is a homozygous deletion of 27 nucleotides (c.2097-2123del). The deleted/altered amino acids exhibited total conservation in zebrafish and partial conservation with 6/9 amino acids in C. elegans conserved. **B**: In the proband (F1) from Family MPS003 there is a homozygous deletion of 3 nucleotides (c.7043delGAG). The deleted amino acids (Glu/E) was conserved in the zebrafish with some degree of functional conservation in C.elegans (D/E).

A novel in-frame deletion of 27 nucleotides (NM_000540.2(RYR1):c.2097_2123del p.(Glu699_Gly707del)) was detected in Family MPS002 of Pakistani origin (Figure 2A).

The family presented at eighteen weeks of pregnancy with a female foetus with bilateral talipes and fixed flexion of the elbows. There was evidence of fetal hydrops on the ultrasound scan including a large cystic hygroma, subcutaneous oedema, ascites also pleural and pericardial effusions. A cardiac ventriculoseptal defect was also suspected. The pregnancy was terminated at 19 weeks of gestation. Post mortem examination revealed no intrauterine growth restriction, but the foetus was considered to be dysmorphic (protuberant eyes, hypertelorism, a flat nose, low set ears), with a complete cleft palate. There was fixed flexion of all the limb joints and a fracture of the proximal left humerus. Pterygia were present between the inferior margin of the mandible and the anterior chest wall and across the elbows. The heart was normal. There were no other congenital abnormalities noted. The brain was structurally and histologically normal. Muscle histopathological analysis is described below.

This candidate *RYR1* c.2097_2123del mutation was present in a homozygous state in two affected foetuses and was found to be heterozygous in both parents. The in-frame deletion was predicted to result in a missense substitution (p.(Glu699Asp)) followed by a deletion of 9 amino acids (GlyTrpGlyGlyAsnGlyValGlyAsp) from the SPRY2 domain of the *RYR1* gene product. All 9 of these deleted amino acids were conserved in vertebrate *RYR1* orthologues including zebrafish and 6 of 9 amino acids were conserved in *C.elegans* (see Figure 2A).

### Clinical and molecular genetic analysis in MPS003 family

**Figure 3:**
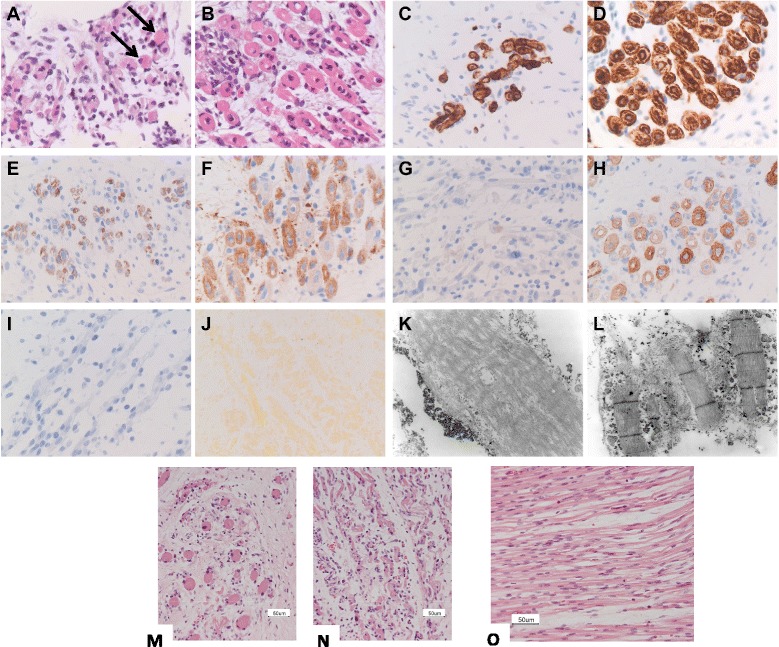
**MPS001: Histological and ultrastructural findings in** ***RYR1*** **-mutant fetal skeletal muscle. (A,B)** Hematoxilin & Eosin stain shows increased fiber size variability in the RYR1-mutant muscle (A, foetus F1) compared to an age-matched control **(B)**. Some RYR1-mutant fibres have intensely eosinophilic cytoplasm (A, arrows). The nuclei are localized centrally in all fibres, compatible with the gestational age. The perinuclear clear halo present in many fibres is an artefact due to formalin fixation. **(C,D)** Labelling against desmin reveals a similar pattern of immunoreactivity and no evident core-like structures in both RYR1-mutant (C, foetus F1) and control tissue **(D)**. **(E-H)** Labelling against the myosin heavy chain fast **(E,F)** and slow **(G,H)** shows that the numbers of myosin fast-positive type II fibres is comparable between the patient (E, foetus F2) and the control **(F)**, whereas myosin slow-positive type I fibres are markedly reduced in RYR1-mutant (G, foetus F2) compared to control muscle **(H)**. **(I)** Labelling for active caspase 3 is negative, excluding apoptosis, also in atrophic RYR1-mutant muscle fibres (foetus F2). **(J)** Alizarin red S staining shows no detectable accumulation of calcium inside the RYR1-mutant muscle fibres (foetus F1). **(K,L)** Ultrastructural analysis reveals profound myofibrillar disarray with disappearance of the Z-bands in the RYR1-mutant muscle fibres (K, foetus F2). By contrast, Z-bands are easily detected in control tissue **(L)**. Magnifications: **(A-J)** 400x; K,L 30000x. (lower panels): histological findings in *RYR1*-mutant (Family MPS003) foetal skeletal muscle, GA 23 weeks. **(M,N)** Hematoxilin & Eosin stain of formalin fixed and paraffin embedded psoas muscle shows loss of fibres with increased fibre size variability and mild fibrosis in the RyR1-mutant muscle **(M,N)** compared to an age-matched control **(O)**.

In a consanguineous family (MPS003) with LMPS, a homozygous in-frame deletion (NM_000540.2(RYR1): c.7043delGAG, p.(Glu2347del) (rs121918596)) was identified in exon 45 in the proband (Figure 2B). This female foetus was the third pregnancy of a consanguineous Palestinian couple. In the pregnancy there was a suggestion of polyhydramnios. On ultrasound examination there were reduced foetal movements and joint contractures were present. The foetus had a cystic hygroma, a hydrothorax, a short neck, a kyphosis and a short trunk due to a scoliosis. The pregnancy was terminated at 23 + 3 weeks of gestation. On examination there was no evidence of intrauterine growth restriction. The foetus had craniofacial anomalies including downslanting palpebral fissures, hypertelorism, a broad nasal bridge, a small mouth and high arched palate, low set ears, a short broad neck and a scoliosis. There were flexion contractures of the shoulders, elbows, wrists, hips, knees and ankles. There were clenched hands but no finger contractures. There was webbing of the axillae, elbows, hips, knees and ankles, and rocker bottom feet. There was a severe scoliosis but no bony abnormalities identified on the radiograph. There was pulmonary hypoplasia. There were no abnormalities of the brain or spinal cord. The muscle in the extremities showed increased fibre size disproportion on microscopy (Figure 3). Cardiac muscle histology was normal.

The karyotype was normal. *CHRNG, CHRNA1, CHRNB1, CHRND, RAPSN* and *DOK7* analysis was normal. Subsequently a fourth pregnancy of a male foetus was terminated at 21 + 3 weeks of pregnancy because of evidence of foetal akinesia. On examination of the foetus no growth restriction was noted. There was a cystic hygroma. The foetus had downslanting palpebral fissures, a broad nasal bridge, a small mouth with a high arched palate. There were contractures of the large and small joints with webbing of the axillae, elbows, hips and knees with pronounced kyphosis, 11 pairs of ribs, but no other bony abnormalities on radiography.

**Figure 4:**
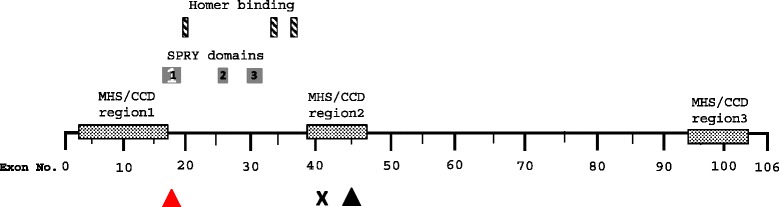
**Location of nonsense mutation in MPS001 (X) and in frame deletions in Family MPS002 (red triangle) and Family MPS003 (black triangle) in relation to exon structure and RYR1 protein domains.** Malignant Hyperthermia/central core disease mutation hot spots shown as stippled boxes, SPRY2 interacting domains 1,2 & 3 as grey boxfibres, Homer binding motifs as hatched boxes.

The foetus had lung hypoplasia and there was a small atrial septal defect. There were no abnormalities of the brain or spinal cord. Skeletal muscle microscopy showed increased fibre disproportion. Immunohistology of the structural proteins (alpha-actin, dystrophin 1,2 and 3, alpha, beta, delta, and gamma sarcoglycan, alpha and beta dystroglycan, dysferlin, caveolin-3, merosin laminin alpha-2 chain, merosin M-chain, myosin heavy chain and spectrin-1) did not identify the pathogenesis of the myopathy (data not shown). It was concluded that the foetus had a non specific myopathy. The couple had one miscarriage and they had an older daughter with a severe undiagnosed metabolic disorder. There were no instances of malignant hyperthermia in the family and the mother had general anaesthesia on several occasions without any problems. The *RYR1* c.7043delGAG candidate mutation was present in both parents in the heterozygous state and there were no other relatives available for analysis. The c.7043delGAG deletion is predicted to result in loss of a glutamic acid residue at codon 2347 in the MHS/CCD hotspot region 2 of the *RYR1* gene product (Figure 4). This residue is conserved in zebrafish and although the amino acid sequence around this residue is divergent in *C. elegans*, the glutamic acid is conserved. Polymorphic variation at codon 2347 was not present in >13,000 *RYR1* alleles reported on the exome variant server (http://evs.gs.washington.edu/EVS), but the c.7043delGAG deletion was previously described, in the heterozygous state, in affected members (total 5 cases) of two unrelated families that presented with malignant hyperthermia [16].

### Histopathological studies

#### Family MPS001

Histopathological analysis was performed on intercostal skeletal muscle from the two foetuses of family MPS001 and two age-matched controls, as this was the only tissue available for both patients. Quantitative histological examination showed marked structural abnormalities in the RYR1-mutant skeletal muscle, including fibre loss, increased fibre size variability and increased endomysial spacing with fibrosis (Figure 3A,B). In agreement with the gestational age, muscle fibres of both patients and controls contained centrally positioned nuclei. Sparse muscle fibres with intense eosinophilic cytoplasm were detected in the patients, but not in the controls (Figure 3A,B) which was not due to calcium accumulation (Figure 3J). Staining against desmin, an intermediate filament protein typically expressed in muscle tissue, revealed no core-like structures (Figure 3C,D). Fibre type distribution, assessed by staining against the slow and fast myosin heavy chain, showed a marked reduction of myosin slow immunoreactivity, suggesting preferential hypotrophy of type I fibres (Figure 3E-H). Labelling for active caspase 3 excluded apoptotic loss of muscle fibres (Figure 3I). Ultrastructural analysis demonstrated hypotrophy with profound myofibrillar disarray and Z-disc loss (Figure 3K,L). Analysis of the spinal cord showed no loss of motor neurons or other pathological signs in the anterior horns and most proximal segments of the motor nerves, supporting the myogenic nature of the histological and ultrastructural findings.

#### Family MPS003

The muscle appearance was striking but non-specific. There was variation in myofibre size with larger hyalinised rounded fibres and smaller rounded atrophic fibres with an apparent increase in fibrous tissue. Fast and slow myosin heavy chains were co-expressed in a proportion of foetal myofibres. Occasional scattered chronic inflammatory cells were confirmed on CD3 and CD20 staining. Additional CD45 staining was interpreted as non-specific. Gomori trichrome staining was negative for nemaline rods and ragged red fibres. The couple had a previous intrauterine death at 23 weeks of gestation of a similarly affected male foetus (Figure 3M-O).

## Discussion

After undertaking genetic linkage studies in a consanguineous family with recurrent FADS/LMPS we mapped a large candidate autozygous region to chromosome 19 and then proceeded to identify a homozygous nonsense mutation in the ryanodine receptor 1 (*RYR1*) gene. *RYR1* encodes the largest known ion channel and the 2.3 MDa homotetrameric ryanodine receptor 1 structure is formed from the 565 kDa RYR1 component proteins. Though the large size (15.3 kb coding sequence in 106 exons) of the *RYR1* gene makes mutation analysis challenging, *RYR1* mutations have previously been described in a variety of human disease phenotypes. Thus initially *RYR1* mutations were described in individuals susceptible to malignant hyperthermia (MHS) [17,18]. *RYR1* mutations associated with MHS are typically heterozygous missense substitutions [19-21]. Subsequently *RYR1* mutations were described in the context of a variety of histological subtypes of congenital myopathies including central core disease, minicore/centronuclear myopathy with external ophthalmoplegia, centronuclear myopathy and congenital fibre-type disproportion [22-24]. Both dominant and recessive forms of *RYR1*-related congenital myopathies have been described and genotype-phenotype correlations have provided insights into likely clinical-functional relationships. Thus, in a large cohort of *RYR1*-associated myopathies, dominant mutations tended to be associated with milder phenotypes whereas recessively inherited cases had an earlier onset and a more severe course [25-27]. In addition, though the age at presentation of recessive *RYR1* myopathies is variable, in a recent series all presented before age 10 years. Whereas *RYR1* mutations in dominantly inherited disease tend to cluster in specific protein domains e.g. C-terminal region (amino acids 4,550-4,940) in central core disease and N-terminal regions (amino acids 35–614 and 2,163-2,458) with MHS, recessively inherited mutations are widely distributed throughout the protein [25,27]. Typically the mutations found in patients with recessively inherited *RYR1*-myopathies are a combination of null mutation with a missense mutation, though two missense mutations can occur. Thus in a series of 118 patients with RYR1-related recessively inherited myopathies from four recent reports [25,27-30], 61.5% of the cases had a truncating/in frame deletion/splice site mutation in combination with a missense mutation and 38.1% harboured two missense mutations. It is therefore striking that we identified a homozygous null mutation in affected individuals from Family MPS001. This observation is consistent with (a) our previous observation for *RAPSN* that homozygosity for a null mutation can cause FADS/LMPS other mutation combinations with only a single null mutation can cause a milder phenotype, (b) that mice homozygous for a *Ryr1* mutation die in the perinatal period with gross abnormalities of skeletal muscle [31] and (c) a history of foetal akinesia may be found with early onset autosomal recessive RYR1-related congenital myopathies [25]. In addition, Romero et al. [32] reported seven foetuses/infants from six unrelated families affected by central core disease in whom there was a history of foetal akinesia [33]. Four cases from three families were found to harbour *RYR1* mutations: three cases (from two families) were compound heterozygotes for *RYR1* missense mutations and in one case only a heterozygous missense mutation was detected. Three of the four cases presented at birth and though in one case the foetus died at 32 weeks gestation (following termination of pregnancy after a previously affected sibling). Thus the phenotype in these cases was less severe than we observed and our findings demonstrate that the association between *RYR1* mutations and foetal akinesia extends to severe early onset lethal FADS and that histopathological evidence of central core disease is not a prerequisite for molecular investigation of *RYR1* in foetal akinesia.

The overall frequency of *RYR1*-related disease in our FADS/LMPS/EVMPS cohort was 4.5% (3/66; 95% CI: 0 to 9.5%) and 8.3% (3/36; 95% CI 0 to 19.5%) in our FADS/LMPS cohort. The case for pathogenicity of the two in frame deletions is supported by their absence from large repositories of genetic variation in control individuals, segregation with disease within the relevant families and evolutionary conservation of the mutated/deleted amino acid residues. RYR1 is a key component of the excitation-coupling process in skeletal muscle such that opening of RYR1 channels result in release of Ca^2+^ from the sarcoplasmic reticulum and initiation of muscle contraction. The novel in-frame deletion of 27 nucleotides (c.2097_2123del p.(Glu699_Gly707del)) detected in Family MPS002 is predicted to result in a missense substitution (p.E699N) followed by a deletion of 9 amino acids (GWGGNGVGD) within the SPRY2 predicted protein-protein interaction motif [32] (highlighted in Figure 4). Previously missense substitutions within or adjacent to this deletion (c.2113G > C; p.Gly705Arg and p.Asp708Asn) have been reported in recessively inherited myopathies [25]. The exon 45 in-frame deletion (c.7043delGAG) identified in Family MPS003 was predicted to result in loss of a glutamic acid residue at codon 2347. A missense mutation at a nearby residue (p.Arg2355Trp) has been reported in both dominantly and recessively inherited myopathies [20] and it is interesting that this deletion was previously described in the heterozygous state, in two unrelated families with malignant hyperthermia [34]. p.Glu2347 is contained within the MHS/CCD mutation hotspot in N-terminal region 2 (stippled box Figure 4), [16]. Though no history of malignant hyperthermia syndrome was reported in Family MPS003, incomplete penetrance is well recognised in malignant hyperthermia and mutation carriers may not have been exposed to trigger events.

Though our findings establish recessive *RYR1* mutations as a cause FADS/LMPS, further work is required to fully establish the frequency of *RYR1* mutations in FADS/LMPS cohorts and to address how novel missense or in-frame deletions/insertions might be reliably interpreted in a clinical diagnostic setting. In a recent review of congenital myopathies treated at a single referral centre, a genetic diagnosis was established in two-thirds of cases and almost 60% of those with a genetic diagnosis had a *RYR1*-related myopathy [27]. Our findings suggest that RYR1-related neuromuscular disease may be a significant cause of FADS/LMPS. Though recessively inherited RYR1-related myopathies have been associated with certain histopathological subtypes such as minicore, centronuclear and congenital fibre-type disproportion myopathies, RYR1 mutations may be associated with other histological subtypes or only nonspecific myopathic features [27]. Extrapolating from these observations, we suggest that, in cases of FADS/LMPS, *RYR1* mutation should be performed as part of a multigene diagnostic strategy (e.g. by second generation sequencing analysis) rather than being specifically targeted to cases with histopathological features that are considered characteristic of a *RYR1*-associated myopathy. The identification of *RYR1* mutations as a cause of familial LMPS/foetal akinesia enables accurate reproductive risk prediction and reproductive options including prenatal diagnosis and pre-implantation diagnosis but also might lead to the identification of relatives at risk of malignant hyperthermia.

## Abbreviations

RYR1: Ryanodine Receptor 1 (skeletal)
MPS: Multiple Pterygium Syndrome
FADS: Foetal Akinesia Deformation Sequence
LMPS: Lethal Multiple Pterygium Syndrome
EVMPS: Escobar-type Multiple Pterygium Syndrome
AChR: Acetylcholine Receptor
CHRNG: Cholinergic Receptor Nicotinic Gamma subunit
CHRNA1: Cholinergic Receptor Nicotinic Alpha1 subunit
CHRND: Cholinergic Receptor Nicotinic Delta subunit
RAPSN: Receptor Associated Protein of the Synapse
DOK7: Docking Protein 7
CMS: Congenital Myesthenic Syndrome
MHS: Malignant Hyperthermia Susceptibility
CCD: Central Core Disease
